# Sound Source Localization Using Non-Conformal Surface Sound Field Transformation Based on Spherical Harmonic Wave Decomposition

**DOI:** 10.3390/s17051087

**Published:** 2017-05-10

**Authors:** Lanyue Zhang, Dandan Ding, Desen Yang, Jia Wang, Jie Shi

**Affiliations:** 1 Science and Technology on Underwater Acoustic Laboratory, Harbin Engineering University, Harbin 150001, China; zhanglanyue@hrbeu.edu.cn (L.Z.); dshyang@hrbeu.edu.cn (D.Y.); wjb562@hrbeu.edu.cn (J.W.); 2 College of Underwater Acoustic Engineering, Harbin Engineering University, Harbin 150001, China; 3 Guizhou Aerospace Institute of Measuring and Testing Technology, Guiyang 550009, China; dingdandan211@163.com

**Keywords:** spherical harmonic wave decomposition, non-conformal sound field surface transformation, reconstruction of sound field, sound source localization

## Abstract

Spherical microphone arrays have been paid increasing attention for their ability to locate a sound source with arbitrary incident angle in three-dimensional space. Low-frequency sound sources are usually located by using spherical near-field acoustic holography. The reconstruction surface and holography surface are conformal surfaces in the conventional sound field transformation based on generalized Fourier transform. When the sound source is on the cylindrical surface, it is difficult to locate by using spherical surface conformal transform. The non-conformal sound field transformation by making a transfer matrix based on spherical harmonic wave decomposition is proposed in this paper, which can achieve the transformation of a spherical surface into a cylindrical surface by using spherical array data. The theoretical expressions of the proposed method are deduced, and the performance of the method is simulated. Moreover, the experiment of sound source localization by using a spherical array with randomly and uniformly distributed elements is carried out. Results show that the non-conformal surface sound field transformation from a spherical surface to a cylindrical surface is realized by using the proposed method. The localization deviation is around 0.01 m, and the resolution is around 0.3 m. The application of the spherical array is extended, and the localization ability of the spherical array is improved.

## 1. Introduction

Sound source localization is the precondition for controlling noise. The main sound sources can be determined through sound source localization, which renders the testimony for making rational means of noise control. The sound field of the sound source surface is reconstructed by using near-field acoustic holography, and the main source is located through the sound field distribution of the sound source surface.

Spherical arrays are superior in sound source localization due to their ability to pick up three-dimensional space sound information. A sound source with an arbitrary incidence angle can be located by using a spherical array [1]. Near-field acoustic holography (NAH) is an acoustic technology of sound source localization and sound field visualization developed in the early 1980s; plane NAH based on space Fourier transformation was proposed by Williams in 1985 [2], and spherical NAH was suggested by Lee in 1996 [3]. The sound source can be located by reconstruction of the spherical surface, which includes the location of the sound sources through spherical NAH by using the measurement data of the spherical array [4]. The spherical NAH does not need the sample in the wave number domain, which avoids wrap-around error. The measurement surface of spherical NAH is a closed surface, which removes the interception of holography aperture [5].

Based on generalized Fourier transformation, as it is difficult to locate the sound source on a cylindrical surface using spherical NAH, the sound field transformation is a conformal surface transformation by using spherical NAH. Boundary element method can be used to realize a non-conformal sound field transform. In fact, boundary element method is suitable for arbitrary shape of sound source surface and holography surface [6,7,8]. However, there is complex interpolation calculation, singular value integration, and nonuniqueness of characteristic wavenumber in the boundary element method, which limit its application in practice. The concept of an orthogonal spherical wave source was established in [9], and the orthogonal spherical wave source boundary element method was researched, which realized the sound field reconstruction and the localization of arbitrary shape and low-frequency sources. Superposition of orthogonal spherical wave source method and boundary point method were compared [10]. A generalized inversion of the transfer matrix must be calculated in the superposition of the orthogonal spherical wave source method. Singular value decomposition of the transfer matrix is needed to realize the spherical wave source superposition. Besides, in order to decrease the effect of noise (which will be amplified in matrix inversion), regularization must be used. The regularization parameters will seriously affect the resolution of the reconstruction sound field, decreasing the stability of the sound field reconstruction method.

In practical engineering applications, it is possible to locate the sound source on a cylindrical surface. With spherical NAH, it is difficult to locate the sound source on a cylindrical surface. Therefore, for this case, the cylindrical array is usually used to locate the sound source. However, the cylindrical array requires a large number of elements, which is bulky and difficult to move and use. In this paper, a non-conformal surface sound field transformation based on spherical harmonic wave decomposition is proposed. Firstly, the coefficients of each order of spherical harmonic function are obtained by using spherical space Fourier transform. Secondly, the transfer matrix of spherical surface and reconstruction cylindrical surface is established. Thirdly, the sound pressure in the wavenumber domain is obtained. Finally, the sound pressure distribution on reconstruction surface can be obtained by using Fourier inverse transform. The image in this paper is the sound pressure amplitude distribution on the reconstructed surface. The sound source will be located through the sound pressure distribution of the reconstruction surface, which includes the localization of the sound source. Compared with the method of orthogonal spherical wave source superposition, the matrix inversion is avoided, and the sensitive factors affecting the sound field transformation are removed by using this method.

## 2. Materials and Methods

### 2.1. Data Model of Spherical Array

**Figure 1:**
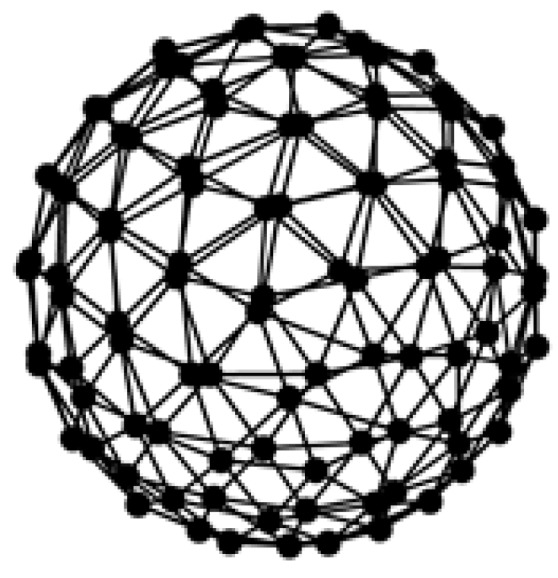
Randomly and uniformly distributed 64-element spherical array.

In this paper, we design a random array of uniformly-distributed spherical arrays to maintain the same target positioning accuracy and multi-objective resolution in different directions. The radius of the spherical array is 0.3 m, and the number of elements is 64. After the radius of the spherical array was determined, 64 points were generated with a random and uniform manner. The position of the 64 points generated at each time was random, however the relative position of the 64 points was uniform. Therefore, the design of the spherical array to ensure the adjacent points connected to the composition of the small area is basically equal [11]. The spherical array is shown in Figure 1.

**Figure 2:**
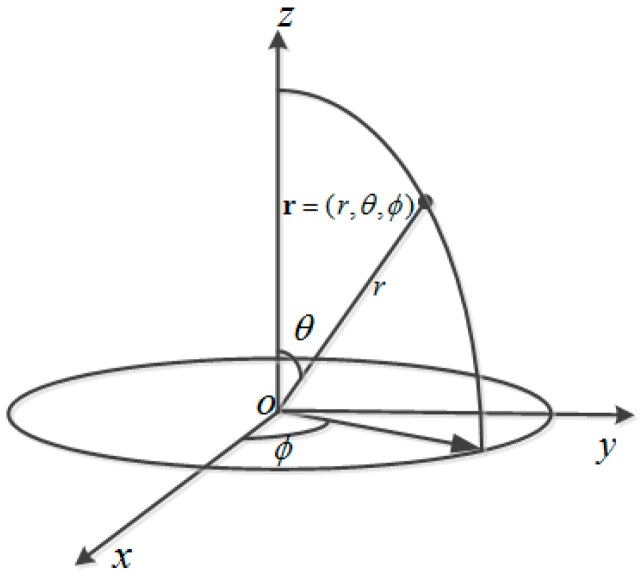
Spherical coordinate system.

The spherical coordinate system is established as shown in Figure 2, with origin of coordinates at the center of the spherical array (0,0,0). The point sound source lies in $\left(x_{0},y_{0},z_{0}\right)$. The coordinates of the *q*th element is $r_{q}=(x_{q},y_{q},z_{q})$ and the sound pressure of the *q*th element is as follows

(1) $$p(r_{q0},t)=P_{0}\frac{1}{r_{q0}}e^{-j(\omega t-kr_{q0})}$$

where $P_{0}$ is the amplitude of sound pressure with distance 1 m, $r_{q0}=\sqrt{{\left(x_{q}-x_{0}\right)}^{2}+{\left(y_{q}-y_{0}\right)}^{2}+{\left(z_{q}-z_{0}\right)}^{2}}$ is the distance of the place of *q*th element to the point sound source, ω is angular frequency, k is wave number, and t is observation time.

There are *Q* microphones composing the spherical array, and the complex sound pressure data of the spherical array is as follows:

(2) $$\begin{array}{ll} P(r,t) & =\left[p_{1}(r_{10},t),\cdots ,p_{q}(r_{q0},t),\cdots ,p_{Q}(r_{Q0},t)\right]\\ & =P_{0}e^{j\omega t}\left[\frac{1}{r_{10}}e^{jkr_{10}},\cdots ,\frac{1}{r_{q0}}e^{jkr_{q0}},\cdots ,\frac{1}{r_{Q0}}e^{jkr_{Q0}}\right] \end{array}$$

There are *K* point sound source incidents on spherical array, and the data of each element is the superposition of the wave of *K* point sound sources. The complex sound pressure data in the frequency domain of the spherical array P(r,θ,ϕ) is obtained by calculating the cross spectrum of the data of each element and the number 1 element

(3) $$P(r,\theta ,\phi )=P_{0}\left[\frac{1}{r_{10}}e^{jkr_{10}},\cdots ,\frac{1}{r_{q0}}e^{jkr_{q0}},\cdots ,\frac{1}{r_{Q0}}e^{jkr_{Q0}}\right]$$

where *θ* and *φ* are the angles indicated in Figure 1. The sound source localization will be obtained by using the complex sound pressure data of the spherical array.

### 2.2. Spherical Harmonic Wave Decomposition

The solution of the Helmholtz equation in spherical coordinates expressed by variables separation method is

(4) $$p(r,\theta ,\phi )=\overset{\infty }{\underset{n=0}{\sum }}\overset{n}{\underset{m=-n}{\sum }}C_{nm}h_{n}^{\left(1\right)}(kr)Y_{n}^{m}(\theta ,\phi )$$

where $C_{nm}$ is the coefficient of the spherical wave decomposition, $h_{n}^{\left(1\right)}(kr)$ is the first kind spherical Hankel function of order n and $Y_{n}^{m}(\theta ,\phi )$ is the spherical harmonic wave function, which is as follows:

(5) $$Y_{n}^{m}(\theta ,\phi )=\sqrt{\frac{(2n+1)(n-m)!}{4\pi (n+m)!}}P_{n}^{m}(\cos\theta )e^{im\phi }$$

where $P_{n}^{m}(•)$ is the associated Legendre function.

When the expansion coefficient $C_{nm}$ is determined, the radiation field is determined. If the sound pressure p(r,θ,ϕ) of the spherical surface with radius r is known, the coefficient of spherical harmonic function $C_{nm}$ can be determined as follows:

(6) $$C_{nm}=\frac{1}{h_{n}^{\left(1\right)}(kr)}\iint_{S}p(r,\theta ,\phi ){\left[Y_{n}^{m}(\theta ,\phi )\right]}^{*}\sin\theta d\theta d\phi$$

where *S* is a spherical surface with radius r, and superscript ∗ represents a complex conjugate. 

The sound pressure in the spectrum domain $P_{nm}$ can be obtained by spherical Fourier transformation of the data picked up by spherical array as follows:

(7) $$P_{nm}=\iint_{S}p(r,\theta ,\phi ){\left[Y_{n}^{m}(\theta ,\phi )\right]}^{*}\sin\theta d\theta d\phi$$

The relation between $P_{nm}$ and $C_{nm}$ is as follows:

(8) $$P_{nm}=C_{nm}h_{n}^{\left(1\right)}(kr)$$

Putting Equation (8) into Equation (4), we get

(9) $$p(r,\theta ,\phi )=\overset{\infty }{\underset{n=0}{\sum }}\overset{n}{\underset{m=-n}{\sum }}P_{nm}Y_{n}^{m}(\theta ,\phi )$$

A discrete special sample for the sound field is performed by using a spherical array to obtain the information of the sound field. The integration of the spherical surface sound pressure changes to the summation of the discrete point sound pressure of each element when calculating the spherical surface wave spectrum $P_{nm}$ as follows:

(10) $$P_{nm}=\sum_{q=1}^{Q}a_{q}p_{q}(r_{q},\theta_{q},\phi_{q}){\left[Y_{n}^{m}(\theta_{q},\phi_{q})\right]}^{*}\;n\in (0,1,\ldots ,N),\;m\in (-n,-n+1,\ldots ,n)$$

where $P_{q}(r_{q},\theta_{q},\phi_{q})$ is the sound pressure of the *q*th element, *Q* is the number of elements of the spherical array, and $\alpha_{q}=4\pi R^{2}/Q$ is the weight coefficient of the special sample point of each element, and *N* is the maximum order of spherical wave needed to be calculate.

### 2.3. Transfer Matrix and Sound Pressure Field Reconstruction

The transfer matrix from equivalent spherical harmonic sound source to reconstruction cylindrical surface is constructed. The reconstruction cylindrical surface is firstly discrete, and the original point of the cylindrical coordinate is coincident to the center point of the spherical array. The radius of the reconstruction cylindrical surface is r and the length is *z*, the discretization for cylindrical surface along the circumference direction and axis direction. The discretization interval is Δϕ in the circumference direction and Δz in the axis direction. The total number of discretization points is *I* × *J.*

Reconstruction cylindrical surface is discretized in cylindrical coordinates and the predicting sound field expression is in spherical coordinates. The transformation of cylindrical coordinates (*r*, *θ*, *φ*) into spherical coordinates (*r’*, *θ’*, *φ’*) is as follows:

(11) $$\left\{ \begin{array}{l} r^{\prime }=\sqrt{r^{2}+z^{2}}\\ \phi^{\prime }=\phi \\ \theta^{\prime }=\arctan(r/z) \end{array}\right.$$

**Figure 3:**
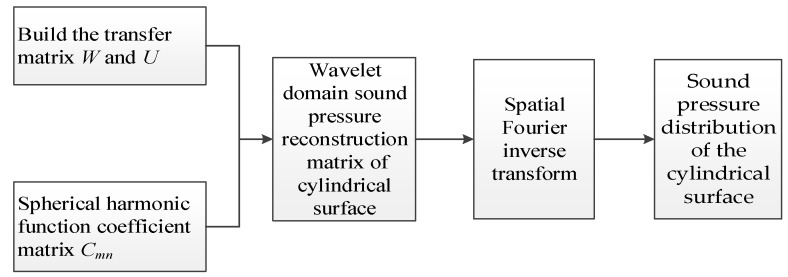
The transformation process.

The transformation process is shown in Figure 3.

The sound pressure distribution of the cylindrical surface is as follows:

(12) $$p(r,\theta ,\phi )=\overset{\infty }{\underset{n=0}{\sum }}\overset{n}{\underset{m=-n}{\sum }}P_{reconstruction}Y_{n}^{m}(\theta ,\phi )$$

$P_{reconstruction}$ is the matrix for sound pressure data of discretization points of the reconstructed cylindrical surface. The specific equation derivations are in Appendix A. The sound pressure distribution of the reconstructed surface including the location of the sound source, and the sound source will be located.

## 3. Results

### 3.1. Simulation Result

#### 3.1.1. Single Sound Source Localization Simulation

Because the array element distribution is uniform on the spherical array, the location of the sound source in different directions is similar. The influence of different frequencies on the sound source localization is great. In this paper, the simulation and experiment of the sound source with different frequency in the same position are carried out. 

**Figure 4:**
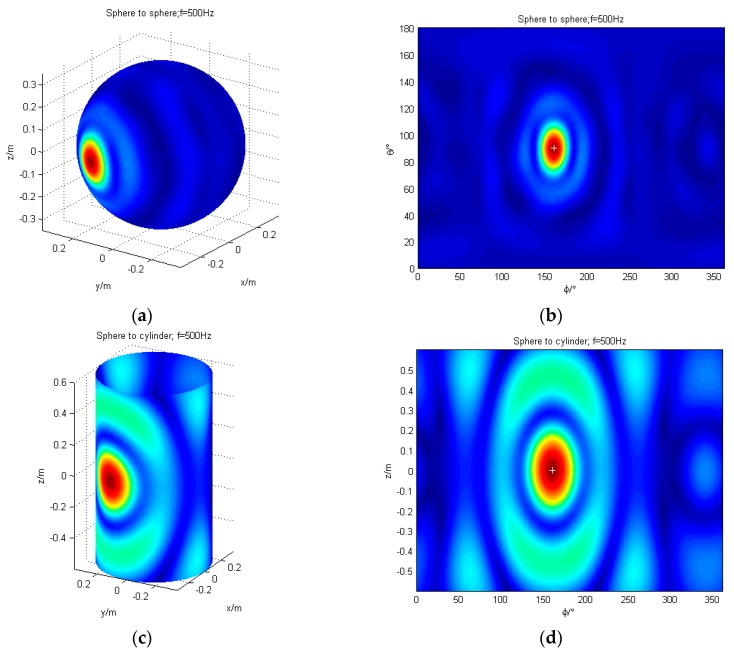
The simulation of single sound source localization (frequency = 500 Hz); (**a**) The 3D map of the reconstruction from sphere to sphere; (**b**) The result of the sphere to the sphere is expanded with *θ* and *φ*; (**c**) The 3D map of the reconstruction from sphere to cylinder; (**d**) The result of the sphere to the cylinder is expanded with *φ* and *z*.

The localization of a single sound source using conformal and non-conformal transformation are simulated. The spherical coordinates of the sound source are (0.35 m, 160°, 90°) and the cylindrical coordinates are (0.35 m, 160°, 0 m). The frequencies of the sound source are 125 Hz, 200 Hz, 315 Hz, 500 Hz, 800 Hz, 1000 Hz, and 1500 Hz, respectively, with Gaussian noise of signal-to-noise ratio (SNR) = 35 dB. Taking 500 Hz as an example, the localization results for a single source are demonstrated in Figure 4. The left portion of each row is a 3D map of the reconstruction surface. The right one is the unfolded map of the reconstruction surface. For comparison, the sound source position is indicated by “+” in the figure.

The validity of non-conformal sound field transformation is proven in Figure 4, which meets the demands of sound source localization for different shapes of sound source. The localization deviation (LD) and localization accuracy are shown in Table 1. Among them, the localization deviation is the difference between the theoretical location of the sound source and the location of the sound source located by algorithm. It is divided into the *θ*-direction and the *φ*-direction or the *z*-direction. The lower the localization deviation, the better. The localization accuracy is the angle of the *φ*-direction corresponding to the sound pressure amplitude falling by 3 dB from the peak. The lower the localization accuracy, the better.

Table 1 shows that after reconstructing to the cylinder, the localization deviation is lower than 0.01 m in the *z*-direction or 1° in the *φ*-direction (the distance on the sphere is 0.005 m) in the frequency band under discussion. The localization accuracy increases as the frequency increases. In the low-frequency band, the low localization accuracy is mainly due to the large cross-sectional area of the sound source when the reconstruction surface is cylindrical.

#### 3.1.2. Two Sound Sources Localization Simulation

**Figure 5:**
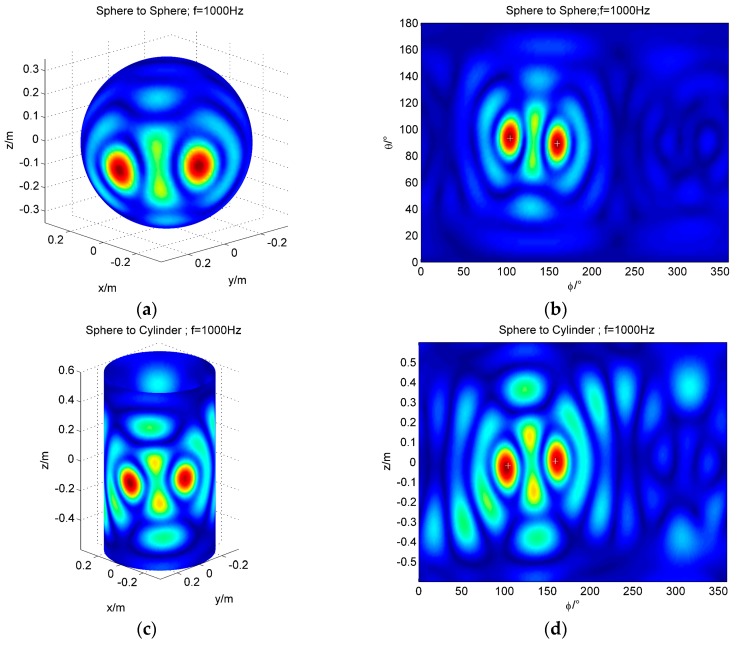
The simulation of two sound sources localization (frequency = 1000 Hz); (**a**) The 3D map of the reconstruction from sphere to sphere; (**b**) The result of the sphere to the sphere is expanded with *θ* and *φ*; (**c**) The 3D map of the reconstruction from sphere to cylinder; (**d**) The result of the sphere to the cylinder is expanded with *φ* and *z*.

Two sound sources localization using conformal and non-conformal sound field transformation are simulated. The spherical coordinates of the two sound sources are, respectively, (0.35 m, 160°, 90°) and (0.35 m, 105°, 93°). The cylindrical coordinates of the two sound sources are, respectively, (0.35 m, 160°, 0 m) and (0.35 m, 160°, −0.02 m). The frequencies of both sound sources are 1000 Hz, with Gaussian noise of SNR = 35 dB. The localization results of conformal and non-conformal sound field transformation are shown in Figure 5. 

Figure 5 shows that two sound sources can be located by using conformal and non-conformal sound field transform. After simulation, we can see that the resolution of the two sources is 50° (the distance on the sphere is 0.3 m). The effectiveness of the non-conformal sound field transformation is verified, and the application domain of the spherical array is extended.

### 3.2. Experiment Result

**Figure 6:**
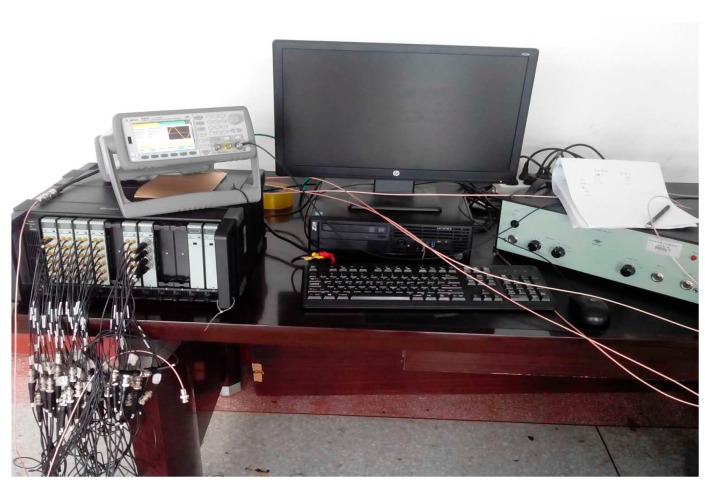
The connection of the field instrument.

**Figure 7:**
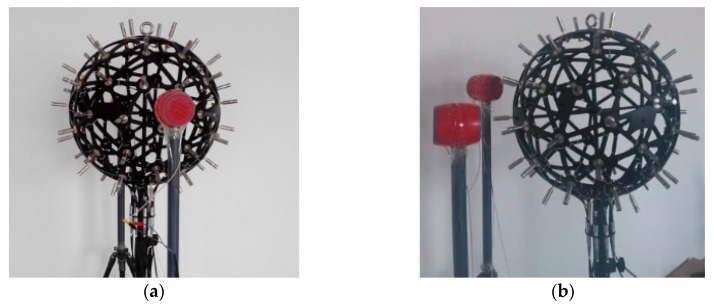
Spherical array and sound sources in experiment; (**a**) One sound source localization experiment; (**b**) Two sound sources localization experiment.

The experimental system was established based on the theoretical research results to test the validity of the proposed sound source localization method. The test environment is an open space that can be regarded as a free sound field, and the test system consists of a transmitter system and a receiving system. The transmitter system consists of a signal source (Model: Agilent33522A, Agilent Technologies, Santa Clara, CA, USA), a power amplifier (Model: B&K2713, Brüel & Kjær. Nærum, Denmark), and an air source. The air source is a cylindrical speaker with a height of 0.095 m and a diameter of 0.08 m. The signal source generates a signal and the amplifier drives small speakers to radiate sound waves. The receiving system consists of a spherical array, a multi-channel signal collector (Model: B&K3660-D, channel: 64, Brüel & Kjær), and a control computer. The microphone array is used to pick up the sound field information, and signal collector is responsible for data acquisition and storage. The test included a single sound source localization test and a two sound source localization test. Figure 6 shows the connection of the field instrument. Figure 7 is for the spherical array measurement of a single source and two sound sources.

#### 3.2.1. Single Sound Source Localization Experiment

**Figure 8:**
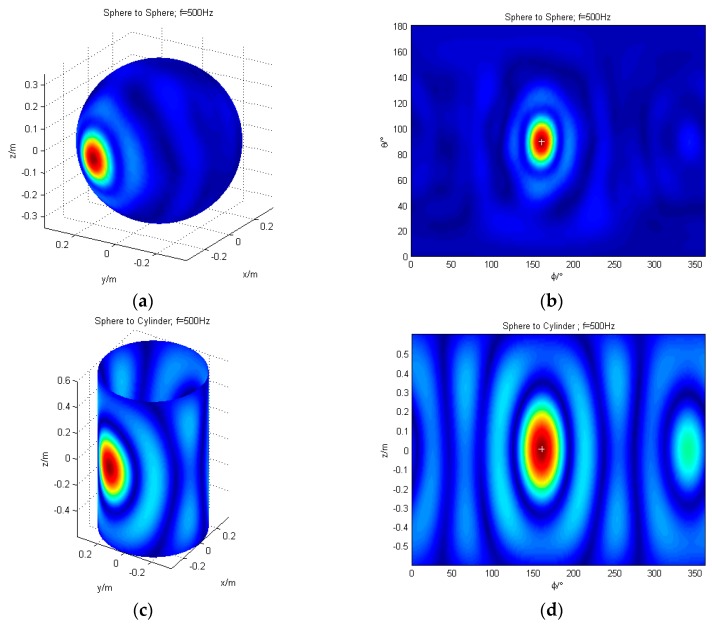
The experiment of single sound source localization (frequency = 1000 Hz); (**a**) The 3D map of the reconstruction from sphere to sphere; (**b**) The result of the sphere to the sphere is expanded with *θ* and *φ*; (**c**) The 3D map of the reconstruction from sphere to cylinder; (**d**) The result of the sphere to the cylinder is expanded with *φ* and *z*.

A speaker which spherical coordinate (0.35 m, 160°, 90°) was placed near the spherical array, and the cylindrical coordinate (0.35 m, 160°, 0 m). The frequencies of sound source were 300 Hz, 400 Hz, 500 Hz, 600 Hz, 700 Hz, 900 Hz, and 1000 Hz. Taking 500 Hz as an example, the localization result is demonstrated in Figure 8.

The results of sound source localization were coincident to the theoretical results. The results of sound source identification were coincident to the theoretical results. The localization deviation (LD) and localization accuracy of single sound source localization experiment are shown in Table 2. 

Table 2 shows that after reconstructing to the cylinder the localization deviation is lower than 0.01 m in the *z*-direction or 2° in the *φ*-direction (the distance on the sphere is 0.01 m). In addition, the experimental results show that the localization of the sound source can be obtained by non-conformal sound field transform.

#### 3.2.2. Two Sound Sources Localization Experiment

**Figure 9:**
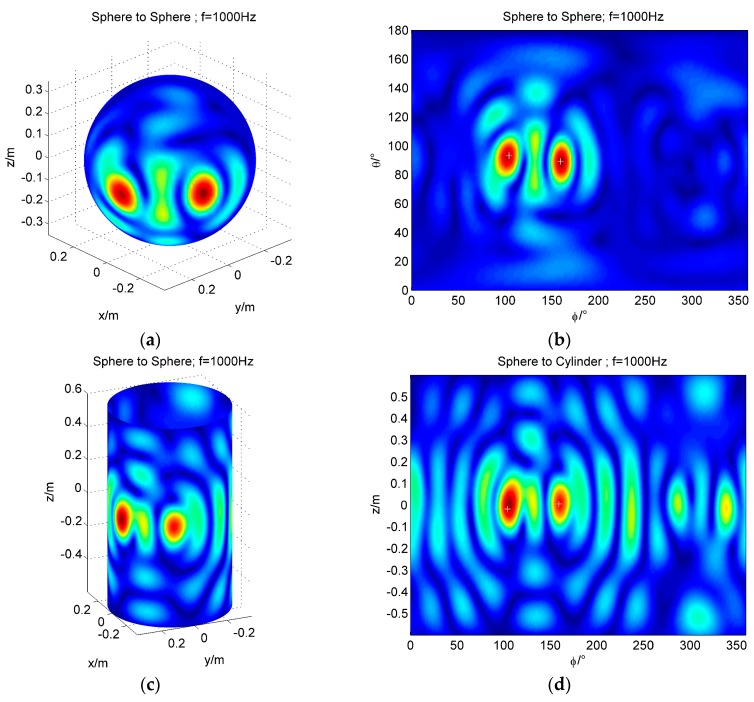
The experiment of two sound sources localization (frequency = 1000 Hz); (**a**) The 3D map of the reconstruction from sphere to sphere; (**b**) The result of the sphere to the sphere is expanded with *θ* and *φ*; (**c**) The 3D map of the reconstruction from sphere to cylinder; (**d**) The result of the sphere to the cylinder is expanded with *φ* and *z*.

The spherical coordinates of two sources were, respectively, (0.35 m, 160°, 90°) and (0.35 m, 105°, 93°), and the cylindrical coordinates were, respectively, (0.35 m, 160°, 0 m) and (0.35 m, 160°, −0.02 m). The frequencies of signals were both 1000 Hz. The localization results by using conformal and non-conformal sound field transformation are shown in Figure 9.

The maximum localization deviation of two sound sources localization by using conformal sound field transformation was 2° in the *φ*-direction (the distance on the sphere is 0.01 m). The maximum localization deviations of two sound sources localization were 2° in the *φ*-direction and 0.01 m in the *z*-direction.

## 4. Discussion

According to the result of sound source simulation and experiment, there was a lower deviation in conformal sound field transformation. On the contrary, the non-conformal sound field transformation by making the transfer matrix had a higher localization deviation. There is no denying that the transfer matrix is the main reason for the localization deviation. The SNR of our experiment was about 30–40 dB. The NAH method needs to be used under the condition of high SNR. When the SNR is lower than 25 dB, it cannot locate the sound source accurately. The non-conformal sound field transformation indeed contributes to locating the sound source on the cylindrical surface. This method can be applied to locate sound sources in aircraft cabins, train cars, and other cylindrical spaces. The localization results are intuitive. However, in the enclosed space, wall reflection sound may have an impact on the localization results. In the future, we can study how to reduce the impact of reflection, and we can study how to transform from sphere to plane to locate the sound source on the plane such as the plane baffle.

## 5. Conclusions

Non-conformal sound field transformation using a spherical array is researched in this paper. The sound field information is picked up by a spherical array. We assume that the equivalent sound source lies in the center of the spherical array. The parameters of different orders for spherical harmonic function are obtained by spherical harmonic wave decomposition. A transfer matrix is constructed between equivalent spherical wave source and reconstruction cylindrical surface. Mode domain sound pressure is obtained by the product of decomposition parameter matrix and transfer matrix. The sound pressure distribution can be obtained by using the Fourier inverse transformation of Mode domain sound pressure. The sound source will be located by using the sound pressure distribution of the reconstruction surface. The high resolution of sound source localization is obtained by using non-conformal sound field transform. The validity of the proposed method is proved by theoretical simulation and experimental results. The application of the spherical array is expanded, and the stability of the sound field reconstruction method is improved.

## Figures and Tables

**Table 1:** The localization deviation and localization accuracy of the simulation of single sound source at (0.35 m, 160°, 90°) with different frequencies. LD: localization deviation.

f/Hz	Sphere to Sphere			Sphere to Cylinder		
	LD in the *φ*-Direction	LD in the *θ*-Direction	Localization Accuracy	LD in the *φ*-Direction	LD in the *z*-Direction	Localization Accuracy
125	0.00°	0.00°	21.50°	0.00°	0.01 m	75.48°
200	0.00°	0.00°	21.39°	0.00°	0.01 m	71.92°
315	0.00°	0.00°	21.21°	0.00°	0.00 m	50.45°
500	0.00°	0.00°	20.89°	0.00°	0.00 m	37.08°
800	0.00°	0.00°	18.92°	1.00°	0.00 m	27.14°
1000	0.00°	0.00°	18.30°	0.00°	0.00 m	20.12°
1500	0.00°	0.00°	21.12°	0.00°	0.00 m	21.14°

**Table 2:** The localization deviation and localization accuracy of the experiment of single sound source at (0.35 m, 160°, 90°) with different frequencies.

f/Hz	Sphere to Sphere			Sphere to Cylinder		
	LD in the *φ*-Direction	LD in the *θ*-Direction	Localization Accuracy	LD in the *φ*-Direction	LD in the *z*-Direction	Localization Accuracy
300	0.00°	0.00°	21.33°	1.00°	0.00 m	43.20°
400	0.00°	0.00°	21.16°	2.00°	0.00 m	41.35°
500	0.00°	0.00°	20.67°	−1.00°	0.00 m	31.97°
600	0.00°	0.00°	20.67°	0.00°	0.01 m	30.79°
700	0.00°	0.00°	20.08°	0.00°	0.01 m	24.57°
900	0.00°	2.00°	19.20°	1.00°	0.01 m	21.37°
1000	0.00°	2.00°	18.41°	−1.00°	0.01 m	17.42°

## Appendix A

The transfer matrixes *W* and *U* are reconstructed as following:

(A1) $$W={\left[\begin{array}{cccccc} h_{n}^{\left(1\right)}(k\sqrt{r^{2}+z_{1}^{2}}) & h_{n}^{\left(1\right)}(k\sqrt{r^{2}+z_{2}^{2}}) & \ldots & h_{n}^{\left(1\right)}(k\sqrt{r^{2}+z_{j}^{2}}) & \ldots & h_{n}^{\left(1\right)}(k\sqrt{r^{2}+z_{J}^{2}}) \end{array}\right]}^{T}$$

(A2) $$U=\left[\begin{array}{cccccc} Y_{n}^{m}\left(\arctan(\frac{r}{z_{1}}),\phi_{1}\right) & Y_{n}^{m}\left(\arctan(\frac{r}{z_{1}}),\phi_{2}\right) & \cdots & Y_{n}^{m}\left(\arctan(\frac{r}{z_{1}}),\phi_{i}\right) & \cdots & Y_{n}^{m}\left(\arctan(\frac{r}{z_{1}}),\phi_{I}\right)\\ Y_{n}^{m}\left(\arctan(\frac{r}{z_{2}}),\phi_{1}\right) & Y_{n}^{m}\left(\arctan(\frac{r}{z_{2}}),\phi_{2}\right) & \cdots & Y_{n}^{m}\left(\arctan(\frac{r}{z_{2}}),\phi_{i}\right) & \cdots & Y_{n}^{m}\left(\arctan(\frac{r}{z_{2}}),\phi_{I}\right)\\ \vdots & \vdots & \cdots & \vdots & \cdots & \vdots \\ Y_{n}^{m}\left(\arctan(\frac{r}{z_{j}}),\phi_{1}\right) & Y_{n}^{m}\left(\arctan(\frac{r}{z_{j}}),\phi_{2}\right) & \cdots & Y_{n}^{m}\left(\arctan(\frac{r}{z_{j}}),\phi_{i}\right) & \cdots & Y_{n}^{m}\left(\arctan(\frac{r}{z_{j}}),\phi_{I}\right)\\ \vdots & \vdots & \cdots & \vdots & \cdots & \vdots \\ Y_{n}^{m}\left(\arctan(\frac{r}{z_{J}}),\phi_{1}\right) & Y_{n}^{m}\left(\arctan(\frac{r}{z_{J}}),\phi_{2}\right) & \cdots & Y_{n}^{m}\left(\arctan(\frac{r}{z_{J}}),\phi_{i}\right) & \cdots & Y_{n}^{m}\left(\arctan(\frac{r}{z_{J}}),\phi_{I}\right) \end{array}\right]$$

where superscript T represents transposition. $P_{reconstruction}$ is the matrix for sound pressure data of discretization points of reconstruction cylindrical surface.

(A3) $$P_{reconstruction}=\left[\begin{array}{cccccc} p(r,\phi_{1},z_{1}) & p(r,\phi_{2},z_{1}) & \cdots & p(r,\phi_{i},z_{1}) & \cdots & p(r,\phi_{I},z_{1})\\ p(r,\phi_{1},z_{2}) & p(r,\phi_{2},z_{2}) & \cdots & p(r,\phi_{i},z_{2}) & \cdots & p(r,\phi_{I},z_{2})\\ \vdots & \vdots & \cdots & \vdots & \cdots & \vdots \\ p(r,\phi_{1},z_{j}) & p(r,\phi_{2},z_{j}) & \cdots & p(r,\phi_{i},z_{j}) & \cdots & p(r,\phi_{I},z_{j})\\ \vdots & \vdots & \cdots & \vdots & \cdots & \vdots \\ p(r,\phi_{1},z_{J}) & p(r,\phi_{2},z_{J}) & \cdots & p(r,\phi_{i},z_{J}) & \cdots & p(r,\phi_{I},z_{J}) \end{array}\right]$$

Let *E* is the 1 × *J* matrix with element 1, we have

(A4) $$P_{reconstruction}=\overset{\infty }{\underset{n=0}{\sum }}\overset{n}{\underset{m=-n}{\sum }}C_{nm}\cdot (W⊗E)\cdot U$$

where · represents dot product and ⊗ represents Kronecker product.

Spherical Fourier inverse transformation of $P_{reconstruction}$ is as follows:

(A5) $$p(r,\theta ,\phi )=\overset{\infty }{\underset{n=0}{\sum }}\overset{n}{\underset{m=-n}{\sum }}P_{reconstruction}Y_{n}^{m}(\theta ,\phi )$$
